# The impact of delayed commencement of adjuvant chemotherapy (eight or more weeks) on survival in stage II and III colon cancer: a national population-based cohort study

**DOI:** 10.18632/oncotarget.17767

**Published:** 2017-05-10

**Authors:** Young Wan Kim, Eun Hee Choi, Bo Ra Kim, Woo-Ah Ko, Yeong-Mee Do, Ik Yong Kim

**Affiliations:** ^1^ Department of Surgery, Division of Colorectal Surgery, Yonsei University Wonju College of Medicine, Wonju, Korea; ^2^ Institute of Lifestyle Medicine, Yonsei University Wonju College of Medicine, Wonju, Korea; ^3^ Department of Internal Medicine, Division of Gastroenterology, Yonsei University Wonju College of Medicine, Wonju, Korea; ^4^ Health Insurance Review & Assessment Service, Seoul, Korea

**Keywords:** colonic neoplasms, adjuvant chemotherapy, survival, mortality

## Abstract

**Background:**

To examine the impact of chemotherapy delay on survival in patients with stage II or III colon cancer and the factors associated with the delay (≥8 weeks) of adjuvant chemotherapy.

**Methods:**

Patients undergoing curative resection and adjuvant chemotherapy in a national population-based cohort were included.

**Results:**

Among 5355 patients, 154 (2.9%) received chemotherapy more than 8 weeks after surgery. Based on a multivariate analysis, the risk factors associated with chemotherapy delay ≥8 weeks were older age [65 to 74 years (hazard ratio [HR]=1.48) and ≥75 years (HR=1.69), p=0.0354], medical aid status in the health security system (HR=1.76, p=0.0345), and emergency surgery (HR=2.43, p=0.0002). Using an 8-week cutoff, the 3-year overall survival rate was 89.62% and 80.98% in the <8 weeks and ≥8 weeks groups, respectively (p=0.008). Independent prognostic factors for inferior overall survival included chemotherapy delay ≥8 weeks (HR=1.49, p=0.0365), older age [65 to 74 years (HR=1.94) and ≥75 years (HR=3.41), p<0.0001], TNM stage III (HR=2.46, p<0.0001), emergency surgery (HR=1.89, p<0.0001), American Society of Anesthesiologists score of 3 or higher (HR=1.50, p<0.0001), and higher transfusion amounts (HR=1.09, p=0.0392).

**Conclusions:**

This study shows that delayed commencement of adjuvant chemotherapy, defined as ≥ 8 weeks, is associated with inferior overall survival in colon cancer patients with stage II or III disease. The delay to initiation of adjuvant chemotherapy is influenced by several multidimensional factors, including patient factors (older age), insurance status (medical aid), and treatment-related factors (emergency surgery).

## INTRODUCTION

Surgical resection is the primary treatment for localized colon cancer. After curative resection, adjuvant chemotherapy is performed to lower the risk of tumor recurrence and metastasis [1]. The oncologic benefits of adjuvant chemotherapy have been proven, and adjuvant chemotherapy is suggested for stage II or III colon cancer patients in the current National Comprehensive Cancer Network (NCCN) guidelines [2]. However, there is no clear consensus on the appropriate timing of adjuvant therapy initiation. In major clinical trials for colon cancer, adjuvant chemotherapy was performed within 6 to 8 weeks after surgery [3, 4].

Time cutoffs defining a delay in adjuvant chemotherapy initiation have varied from one month to three months in previous studies [5-19], and a time delay in chemotherapy has been reported to adversely influence [5, 7, 10, 11, 14-17, 19] or not influence the oncologic outcome (Table 1) [6, 8, 9, 12, 13, 18]. It is still controversial whether a delay in chemotherapy negatively influences oncologic outcomes. Thus, it may be more ethical to perform a retrospective study rather than a prospective study to investigate the impact of the delayed commencement of chemotherapy on survival.

**Table 1:** Literature review of time to adjuvant chemotherapy for colon cancer

Author	Data source	N	Site	TNM	Chemotherapy regimen	Survival (%)
Hershman [5] 2006	SEER-Medicare, US	4382	Colon	III	5FU	OS <1 months, 1-2 months, 2-3 months*, >3 months*
Andre [6] 2007	Multicenter, Europe	905	Colon	II, III	5FU	OS ≤35 vs. >35 days (77% vs. 76%**)
Berglund [8] 2008	Multicenter, Sweden	231	Colon	III	5FU	OS ≤56 vs. >56 days**
Zaig-Owens [13] 2009	Massachusetts Cancer Registry, US	3006	Colon	II, III	NA	OS ≤45 vs. 45 days**
Bayraktar [7] 2009	Multicenter, US	186	Colon	II, III	5FU	OS ≤60 vs. >60 days (78.6% vs. 56.7%*)
Czaykowski [9] 2011	Multicenter, Canada	345	Colon	III	5FU	OS ≤56 vs. >56 days** (≤66 years*)
Lima [10] 2011	Alberta Cancer Registry, Canada	1053	Colon	III	NA	OS ≤12 vs. >12 weeks*
Yu [12] 2013	Multicenter, US	102	Colon	III	Oxaliplatin	Time to recurrence ≤12 vs. >12 weeks** (a trend toward)
Xu [11] 2014	SEER-Medicare, US	4209	Colon	II	5FU	OS <3 vs. ≥3 months*
Massarweh [16]2014	National Cancer Data Base, US	51331	Colon	III	NA	OS ≤2 months (69.8%) 2-4 months (62%)* 4-6 months (51.4%)*
Bos [14] 2015	Netherlands Cancer Registry	6620	Colon	III	5FU, oxaliplatin (incomplete)	OS ≤8 vs. >8 weeks*
Nachiappan [17] 2015	Hospital Episode Statistics, UK	18306	Colon	NA	NA	OS ≤8 vs. >8 weeks*
Klein [15] 2015	Danish Colorectal Cancer Group	1827	Colon	III	NA	OS ≤8 vs. >8 weeks*
Peixoto [18] 2015	British Columbia Cancer Agency (BCCA) Gastrointestinal Cancers Outcomes Database	635	Colon	III	Oxaliplatin	CSS ≤8 vs. >8 weeks**
Sun [19] 2015	National Cancer Data Base, US	7794	Colon	II, III	NA	OS ≤44 vs. >44 days*
Current study 2016	Korean Health Insurance Review and Assessment Service	5355	Colon	II, III	5FU, oxaliplatin	OS <8 vs. ≥8 weeks*

TNM, tumor-node-metastasis; FU, fluorouracil; OS, overall survival; SEER, Surveillance Epidemiology and End Results; NA, not available

^*^ P<0.05

Since 2011, the Korean Health Insurance Review and Assessment Service (HIRA), which is a government agency, has mandated the submission of treatment data for new colon cancer patients, including chemotherapy timing, and has recommended the initiation of adjuvant chemotherapy within 8 weeks after surgery. Using these national population-based cohort data, this study aimed to investigate the impact of delayed chemotherapy on overall survival and factors associated with the delayed commencement of adjuvant chemotherapy after colon cancer surgery.

## RESULTS

### Chemotherapy regimens according to TNM stage

Among the 5355 patients with stage II (n=2022) and III (n=3333) disease, the adjuvant chemotherapy regimens were fluoropyrimidine-based (n=1424, 70.43%) and oxaliplatin-based (n=598, 29.57%) in stage II patients and fluoropyrimidine-based (n=872, 26.16%) and oxaliplatin-based (n=2461, 73.84%) in stage III patients (Table 2). A total of 154 (2.88%) patients received chemotherapy 8 weeks or more after surgery.

**Table 2:** Chemotherapy regimens according to TNM stage

Regimens	TNM II (N=2022)	TNM III (N=3333)	P-value
Fluoropyrimidine-based	1424 (70.43%)	872 (26.16%)	<0.0001
Oxaliplatin-based	598 (29.57%)	2461 (73.84%)	

TNM, tumor-node-metastasis

### Factors associated with the delay of adjuvant chemotherapy according to 2-week time intervals

Four subgroups were compared using 2-week time intervals (<4 weeks, 4-6 weeks, 6-8 weeks, and ≥8 weeks groups). As the delay in chemotherapy initiation increased, older age based on a continuous variable (p=0.0039) and age subgroups (p=0.0006), medical aid (vs. health insurance, p=0.0165), stage II disease (vs. stage III, p=0.0161), emergency surgery (vs. elective surgery, p<0.0001), examination of <12 lymph nodes (vs. 12 or more nodes, p=0.0002), and use of fluoropyrimidine-based regimens (vs. oxaliplatin-based, p<0.0001) showed a gradual increasing trend (Table 3).

**Table 3:** Factors associated with the delay of adjuvant chemotherapy according to 2-week time intervals (n=5355)

Variables		<4 weeks	4-6 weeks	6-8 weeks	≥8 weeks	P-value
		N=2695	N=2124	N=382	N=154	
Hospital type	Tertiary referral hospital	2492 (92.47%)	1945 (91.57%)	366 (95.81%)	149 (96.75%)	0.0066
	General hospital (≥100 beds)	202 (7.5%)	179 (8.43%)	16 (4.19%)	5 (3.25%)	
	District hospital (<100 beds)	1 (0.04%)	0 (0%)	0 (0%)	0 (0%)	
Age (years)	Mean±SD	62.72±11.23	62.73±10.92	64.18±11.68	65.29±10.52	0.0039
Age subgroups (years)	<65	1437 (53.32%)	1127 (53.06%)	180 (47.12%)	60 (38.96%)	0.0006
	65-74	852 (31.61%)	708 (33.33%)	130 (34.03%)	59 (38.31%)	
	≥75	406 (15.06%)	289 (13.61%)	72 (18.85%)	35 (22.73%)	
Sex	Male	1625 (60.3%)	1227 (57.77%)	238 (62.3%)	97 (62.99%)	0.1462
	Female	1070 (39.7%)	897 (42.23%)	144 (37.7%)	57 (37.01%)	
National health security system	Health insurance	2549 (94.58%)	1983 (93.36%)	355 (92.93%)	137 (88.96%)	0.0165
	Medical aid	146 (5.42%)	141 (6.64%)	27 (7.07%)	17 (11.04%)	
TNM	II	1000 (37.16%)	796 (37.48%)	145 (37.96%)	77 (50%)	0.0161
	III	1691 (62.84%)	1328 (62.52%)	237 (62.04%)	77 (50%)	
Emergency	Yes	170 (6.31%)	126 (5.93%)	37 (9.79%)	22 (14.29%)	<0.0001
	No	2523 (93.69%)	1997 (94.07%)	341 (90.21%)	132 (85.71%)	
ASA score	1,2	2342 (89.29%)	1855 (88.84%)	326 (87.17%)	133 (87.5%)	0.6045
	3,4	281 (10.71%)	233 (11.16%)	48 (12.83%)	19 (12.5%)	
Lymph nodes retrieved (number)	<12	280 (10.65%)	150 (7.22%)	43 (11.75%)	18 (12.16%)	0.0002
	≥12	2348 (89.35%)	1927 (92.78%)	323 (88.25%)	130 (87.84%)	
Comorbidity	(+)	1924 (71.39%)	1577 (74.25%)	284 (74.35%)	117 (75.97%)	0.1029
Transfusion (units)	Mean±SD	0.04±0.49	0.07±0.72	0.05±0.31	0.14±0.78	0.0862
Chemotherapy regimen	Fluoropyrimidine-based	1181 (43.82%)	856 (40.30%)	170 (44.50%)	89 (57.79%)	<0.0001
	Oxaliplatin-based	1514 (56.18%)	1268 (59.70%)	212 (55.50%)	65 (42.21%)	

SD, standard deviation; TNM, tumor-node-metastasis; ASA, American Society of Anesthesiologists

### Factors associated with the delay of adjuvant chemotherapy using an 8-week cutoff

Based on the multivariate analysis, the factors associated with chemotherapy delay (≥8 weeks) were older age (hazard ratio (HR) = 1.48 in 65-74-year-old patients and 1.69 in ≥75-year-old patients, p=0.0354), medical aid (HR=1.76, p=0.0345), and emergency surgery (HR=2.43, p=0.0002) (Table 4).

**Table 4:** Factors associated with adjuvant chemotherapy delay using an 8-week cutoff (n=5355)

Variables	Univariate analysis			Multivariate analysis		
		<8 weeks N=5201	≥8 weeks N=154	P-value	OR (95% CI)	P-value
Hospital type	Tertiary referral hospital	4803 (92.35%)	149 (96.75%)	0.0698	NA	
	General hospital (≥100 beds)	397 (7.63%)	5 (3.25%)			
	District hospital (<100 beds)	1 (0.02%)	0 (0%)			
Age (years)	Mean±SD	62.83±11.14	65.29±10.52	0.0069	NA	
Age subgroups (years)	<65	2744 (52.76%)	60 (38.96%)	0.0013	1	0.0354
	65-74	1690 (32.49%)	59 (38.31%)		1.48(1.03-2.15)	
	≥75	767 (14.75%)	35 (22.73%)		1.69(1.08-2.65)	
Sex	Male	3090 (59.41%)	97 (62.99%)	0.373	NA	
	Female	2111 (40.59%)	57 (37.01%)			
National health security system	Health insurance	4887 (93.96%)	137 (88.96%)	0.0111	1	0.0345
	Medical aid	314 (6.04%)	17 (11.04%)		1.76(1.04-2.97)	
TNM	II	1945 (37.40%)	77 (50%)	0.0014	1.42(0.99-2.03)	0.0512
	III	3256 (62.60%)	77 (50%)		1	
Emergency	Yes	333 (6.41%)	22 (14.29%)	0.0001	2.43(1.52-3.90)	0.0002
	No	4861 (93.59%)	132 (85.71%)		1	
ASA score	1,2	4523 (88.95%)	133 (87.5%)	0.5754	NA	
	3,4	562 (11.05%)	19 (12.5%)			
Lymph nodes retrieved (number)	<12	473 (9.33%)	18 (12.16%)	0.2443	NA	
	≥12	4598 (90.67%)	130 (87.84%)			
Comorbidity	(+)	3785 (72.77%)	117 (75.97%)	0.3788	NA	
Transfusion (units)	Mean±SD	0.05±0.59	0.14±0.78	0.168	NA	

OR, odds ratio; CI, confidence interval; NA, not applicable; SD, standard deviation; TNM, tumor-node-metastasis; ASA, American Society of Anesthesiologists

### Overall survival rates according to the delay of adjuvant chemotherapy

**Figure 1:**
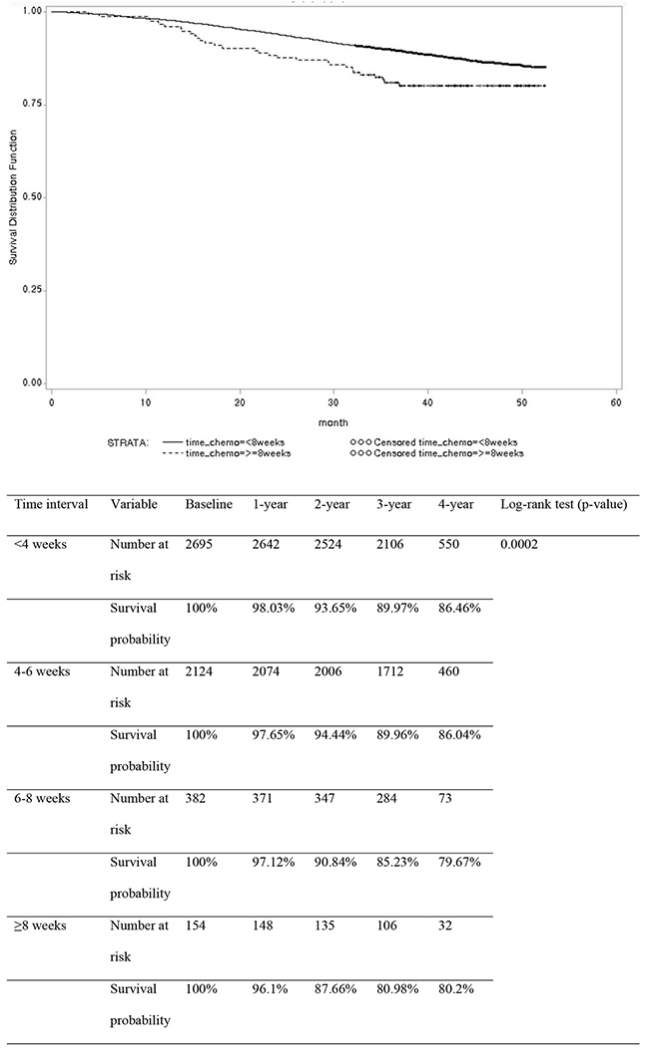
Overall survival rates in patients with stage II or III colon cancer according to chemotherapy delay using 2-week time intervals (n=5355)

**Figure 2:**
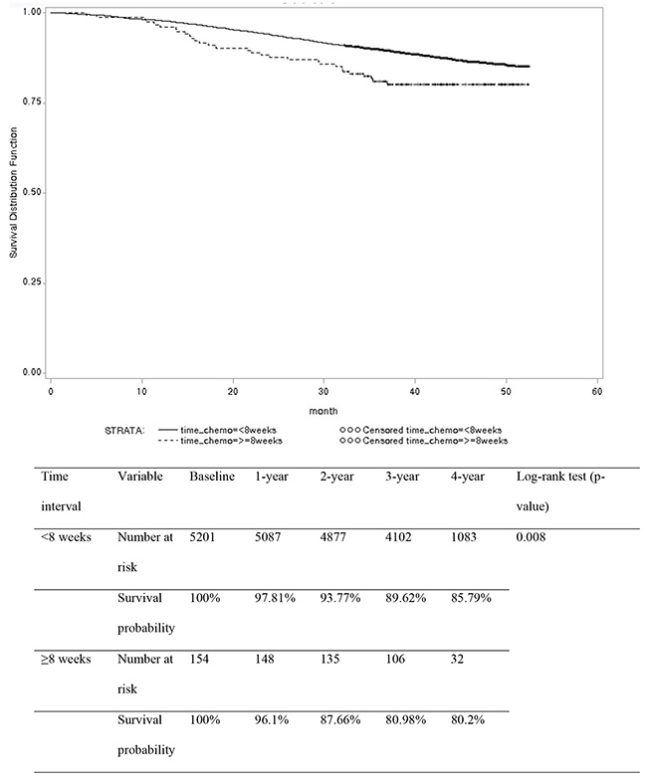
Overall survival rates in patients with stage II or III colon cancer according to chemotherapy delay using an 8-week cutoff (n=5355)

Using 2-week time intervals, the 3-year overall survival rate was 89.97%, 89.96%, 85.23%, and 80.98% in the <4 weeks, 4-6 weeks, 6-8 weeks, and ≥8 weeks groups, respectively (p=0.0002) (Figure 1). Using an 8-week cutoff, the 3-year overall survival rate was 89.62% and 80.98% in the <8 weeks and ≥8 weeks groups, respectively (p=0.008) (Figure 2).

### Prognostic factors for overall survival using Cox proportional hazard modeling

Adverse prognostic factors for overall survival were time to adjuvant chemotherapy (≥8 weeks: HR=1.49, p=0.0365), older age (HR=1.94 in 65-74-year-olds and 3.41 in ≥75-year-olds, p<0.0001), TNM stage III (HR=2.46, p<0.0001), emergency surgery (HR=1.89, p<0.0001), American Society of Anesthesiologists (ASA) score of 3 or higher (HR=1.50, p<0.0001), and a greater transfusion volume (HR=1.09, p=0.0392) (Table 5).

**Table 5:** Prognostic factors for overall survival in patients with stage II or III colon cancer who received adjuvant chemotherapy (n=5355)

	Univariate analysis			Multivariate analysis	
		HR (95% CI)	P-value	HR (95% CI)	P-value
Time to adjuvant chemotherapy	<8 weeks	1	0.0087	1	0.0365
	≥8 weeks	1.63(1.13-2.35)		1.49(1.03-2.15)	
Age subgroups (years)	<65	1	<0.0001	1	<0.0001
	65-74	2.01(1.68-2.40)		1.94(1.61-2.34)	
	≥75	3.65(3.02-4.42)		3.41(2.78-4.18)	
Sex	Male	1.06(0.91-1.23)	0.4963	NA	
	Female	1			
National health security system	Health insurance	1	0.0054	1	0.2848
	Medical aid	1.47(1.12-1.92)		1.17(0.88-1.54)	
TNM	II	1	<0.001	1	<0.0001
	III	2.44(2.03-2.94)		2.46(2.03-2.98)	
Emergency	Yes	2.03(1.61-2.56)	<0.0001	1.89(1.46-2.45)	<0.0001
	No	1		1	
ASA score	1,2	1	<0.0001	1	<0.0001
	3,4	2.05(1.69-2.49)		1.50(1.22-1.84)	
Lymph nodes retrieved (number)	<12	1.38(1.09-1.73)	0.0067	1.25(0.99-1.58)	0.0656
	≥12	1		1	
Comorbidity	(+) vs. (−)	1.18(0.99-1.40)	0.0685	NA	
Transfusion (units)		1.10(1.01-1.19)	0.0222	1.09(1.00-1.18)	0.0392

HR, hazard ratio; CI, confidence interval; NA, not applied; TNM, tumor-node-metastasis; ASA, American Society of Anesthesiologists

## DISCUSSION

The major finding of this study was that the delayed commencement (≥8 weeks) of chemotherapy negatively influenced overall survival. Based on the multivariate Cox proportional hazard model, the delayed initiation of chemotherapy was an unfavorable prognostic factor for overall survival. These findings suggest that the timely initiation of chemotherapy is oncologically important for patients with stage II or III disease. Factors associated with the delay of chemotherapy were patient factors (older age), insurance status (medical aid), and treatment-related factors (emergency surgery).

The underlying mechanisms associated with worse outcomes in patients undergoing delayed chemotherapy initiation have been poorly understood, although potential hypotheses have been suggested [20]. In animal models, it has been hypothesized that primary tumor removal may promote metastatic tumor growth by the conversion of resting cells in the G0 phase to the proliferative phase [21], and surgery can stimulate tumor cell growth by enhancing the release of growth factors during the subsequent healing process [22]. In breast cancer patients, it has been suggested that surgery can induce the angiogenic potential of micrometastases and awaken distant dormant micrometastases [23]. Surgical stress can impair immune function by suppressing the function of cytotoxic T cells and natural killer cells and induce the proliferation of micrometastases [24]. These findings indicate that earlier exposure to chemotherapeutic agents following surgery may be beneficial in terms of reducing the risk of recurrence and preventing the development of metastases.

### Oncologic outcomes according to chemotherapy delay

Whether the delayed commencement of chemo-therapy affects oncologic outcomes is still controversial. No randomized clinical trial with respect to the timing of chemotherapy has been performed in patients with colon cancer. Upon review of the literature, all studies are retrospective series and study subjects are heterogeneous in terms of cancer stage, chemotherapeutic agents, definition of chemotherapy delay, and survival parameters. With regard to TNM stage, studies investigated stage II [11] or III only [5, 8-10, 12, 14-16, 18] or both stage II and III disease [6, 7, 13, 19], and one study did not address cancer stage [17]. We included patients with stage II or III disease, as current NCCN and Korean clinical practice guidelines recommend adjuvant chemotherapy for stage II and III colon cancer. Regarding chemotherapy regimens, 5-fluorouracil-based [5-9, 11], oxaliplatin-based [12, 18], or both 5-fluorouracil and oxaliplatin-based regimens [14] were used, and some studies did not address specific chemotherapy regimens [10, 13, 15-17, 19]. Our cohort included a significant number of patients undergoing oxaliplatin-based chemotherapy, as current guidelines recommend this regimen for stage II disease with high-risk features or stage III disease. This study consisted of patients treated from 2011 to 2012 to reflect current clinical practice. Time delays of 1-3 months [5], 35 days [6], 44 days [19], 45 days [13], 56 days [8, 9], 8 weeks [14, 15, 17, 18], 60 days [7], 12 weeks [10, 12], 3 months [11], and 2-6 months [16] were used. We initially categorized four subgroups (<4 weeks, 4-6 weeks, 6-8 weeks, and ≥8 weeks) and ultimately used an 8-week cutoff, which is recommended by the Korean government’s health service (HIRA). In this study, only 2.88% of the study population received chemotherapy 8 weeks or more after surgery, which reveals that the government regulation is effective regarding the timing of adjuvant chemotherapy. In terms of survival parameters, overall survival was investigated in most studies [5-11, 13-17, 19], but the time to recurrence [12] or cancer-specific survival [18] were rarely investigated. We analyzed overall survival, as we could not identify the specific cause of death for this study population.

The delayed commencement of chemotherapy did not compromise survival in some studies [6, 8, 9, 12, 13, 18]; however, in other studies, a delay negatively influenced survival [5, 7, 10, 11, 14-17, 19]. In this study, using 2-week time intervals, 3-year overall survival rates showed a gradual decreasing pattern according to time delay (89.97%, 89.96%, 85.23%, and 80.98% in the <4 weeks, 4-6 weeks, 6-8 weeks, and ≥8 weeks groups, respectively). Using an 8-week cutoff, delayed chemotherapy (≥8 weeks) compromised 3-year overall survival rates (80.98%) compared with timely administration (89.62% in the <8 weeks group). The delayed initiation of chemotherapy was an independent prognostic factor for adverse overall survival.

### Factors associated with the delay of adjuvant chemotherapy

Diverse factors, such as older age, black race, unmarried status, presence of postoperative complications, prolonged postoperative recovery, emergency surgery, severe comorbid conditions, advanced tumor grade, and institutional time delay between departmental consultations, are associated with delayed adjuvant chemotherapy [5, 25-28]. In this study, using 2-week time intervals (<4 weeks, 4-6 weeks, 6-8 weeks, and ≥8 weeks groups), the delayed initiation of chemotherapy was associated with an increasing trend toward older age, medical aid, stage II disease, emergency surgery, examination of <12 lymph nodes, and use of fluoropyrimidine-based regimens. The relationship between emergency surgery and lymph node count can be explained by the fact that emergency surgery is associated with a low lymph node yield [29]. Fluoropyrimidine-based regimens can be administered to stage II and III patients; however, stage II with high-risk features and stage III patients are primarily recommended to receive oxaliplatin-based regimens. Thus, we could speculate that if the initiation of chemotherapy was delayed over 8 weeks, fluoropyrimidine-based regimens were favored.

Independent risk factors associated with chemotherapy delay (≥8 weeks) were older age, medical aid, and emergency surgery. The risk factors identified in the present study for a chemotherapy delay, such as older age, low income status in terms of medical aid, and emergency surgery, are similar to findings from previous studies. However, the presence of comorbidity based on the Charlson comorbidity index and ASA score was not significant. This study cohort included all Koreans; however, we were unable to obtain certain data, such as marital status and the presence of postoperative complications, due to the nature of national population-based data.

This study is limited by its retrospective design. In addition, data regarding stage II disease with high-risk features, chemotherapy dose reduction, and cancer-related deaths were not available. Another limitation is that the number of patients receiving delayed chemotherapy was small (n=154), which may have introduced a confounding bias. However, this study has several strengths. First, most earlier studies were conducted in Western countries. This study cohort yielded a large quantity of data from a Korean population. Moreover, the data included 21 structured items from a national project conducted by a government health service agency seeking to monitor and evaluate quality of colon cancer care. Second, the study results were derived from chemotherapeutic agents currently being used according to current guidelines for colon cancer treatment.

In summary, this national population-based cohort study shows that delayed commencement of adjuvant chemotherapy, defined as ≥8 weeks, is associated with inferior overall survival in colon cancer patients with stage II or III disease. The delay to initiation of adjuvant chemotherapy is influenced by multidimensional factors, including patient factors (older age), insurance status (medical aid), and treatment-related factors (emergency surgery). Unfortunately, these factors are difficult to modify during colon cancer care. Our findings indicate further studies will be necessary when considering the time to commencement of chemotherapy as a quality indicator for colon cancer care.

## MATERIALS AND METHODS

### Patients

This retrospective study was performed using a national population-based cohort and followed the Strengthening the Reporting of Observational Studies in Epidemiology (STROBE) guidelines [30]. This study was approved and informed consent was waived by the ethics review committee of the HIRA (Seoul, South Korea) and the Institutional Review Board of Wonju Severance Christian Hospital (YWMR-14-5-089).

Since 2011, all hospitals in Korea requesting reimbursement for colorectal cancer treatment have been required to submit 21 items of medical data on all newly diagnosed colorectal cancer patients over 18 years of age. This project was named ‘monitoring and evaluation of quality of colon cancer care’. The diseases evaluated were C18 (malignant neoplasm of the colon), C19 (malignant neoplasm of the rectosigmoid junction), and C20 (malignant neoplasm of the rectum) based on the International Statistical Classification of Diseases and Related Health Problems (ICD)-10 version. The 21 items evaluated in detail were the following: 1, presence of a specialized cancer care team; 2, record of preoperative pain score; 3, record of preoperative family history; 4, appropriate preoperative examination (serum carcinoembryonic antigen (CEA), abdomino-pelvic computed tomography scan, upper gastrointestinal endoscopy, colonoscopy, and pelvic magnetic resonance imaging for rectal cancer); 5, operation record for completeness of resection (R0, R1, or R2); 6, postoperative serum CEA within 3 months after surgery; 7, quality of pathological reports; 8, number of lymph nodes examined (more than 12 nodes); 9, quality of medical records related to cancer treatment; 10, ostomy education; 11, whether adjuvant chemotherapy was administered in stage I disease; 12, commencement of adjuvant chemotherapy within 8 weeks after surgery; 13, education on adjuvant chemotherapy plan; 14, use of a flow sheet to record schedule and dose of adjuvant chemotherapy; 15, whether recommended chemotherapy regimens were administered according to colorectal cancer treatment guidelines; 16, use of antiemetics during chemotherapy; 17, postoperative radiation therapy for rectal cancer; 18, preoperative concurrent chemoradiation therapy for rectal cancer; 19, length of hospital stay; 20, treatment cost; and 21, in-hospital mortality. The HIRA collected the aforementioned data to improve the quality of cancer care at the national level.

This study cohort consisted of a total of 5355 patients with stage II or III colon cancer diagnosed between January 1, 2011, and December 31, 2012, who underwent curative resection and completed adjuvant chemotherapy at all hospitals registered in the Korean HIRA. Eligibility criteria included patients with histologically confirmed colon adenocarcinoma and patients over the age of 18 years. The exclusion criteria were patients with stage I or IV diseases, patients undergoing incomplete resection (R2, macroscopic residual disease) or palliative non-resectional procedures, patients not receiving adjuvant chemotherapy, or patients with rectal cancer.

### Study objectives

The primary objective was to evaluate the impact of delayed chemotherapy (eight or more weeks) on overall survival. The secondary objective was to evaluate factors associated with the delayed commencement of adjuvant chemotherapy after colon cancer surgery.

### Adjuvant chemotherapy and follow-up

After adequate recovery following curative resection, all patients with stage II or III disease were recommended to receive chemotherapy according to the Korean clinical practice guidelines for colon and rectal cancer v.1.0 [31, 32]. Chemotherapy regimens included fluoropyrimidine (fluorouracil with folinic acid, capecitabine) alone or in combination with oxaliplatin (FOLFOX). Patients with stage II disease who were at high risk for recurrences (T4, poor histological grade, peritumoral lymphovascular involvement, bowel obstruction at presentation, T3 lesions with localized perforation or close, indeterminate, or positive resection margins, or perineural invasion) were recommended to undergo oxaliplatin-containing regimens.

Patient follow-up was continued until death or August 31, 2015. Mortality data were obtained from the national health insurance service (Seoul, Korea). The Korean national health insurance program covers all 51.6 million South Koreans, and the national health security system provides health insurance and medical aid based on economic status according to income level. The median follow-up period was 42.2 months (mean ± standard deviation: 39.2 ± 11.7 months).

### Outcome measures

The time to adjuvant chemotherapy initiation was defined as the time interval from the date of surgery to the date of the commencement of adjuvant chemotherapy. The delayed commencement of adjuvant chemotherapy was defined as the start of chemotherapy 8 or more weeks after surgery. The presence of comorbidity was defined as the presence of one of any medical condition presented in the Charlson comorbidity index [33]. The quantity of blood transfusion was calculated during the hospital stay after the index surgery.

### Statistical analysis

All statistical analyses were performed using SAS version 9.2 (SAS Institute Inc., Cary, NC, USA). Categorical variables are presented as frequencies and percentages and were compared by the chi-square test or Fisher’s exact test as appropriate. Continuous variables are presented as means and standard deviations and were analyzed by the two-sample *t*-test and analysis of variance (ANOVA). Factors associated with the delay of chemotherapy were identified by logistic regression analysis. Survival analysis was performed by the Kaplan-Meier method with log-rank tests and the Cox proportional hazard model. A *p*-value <0.05 was considered statistically significant.

## Abbreviations

NCCN: National Comprehensive Cancer Network
ASA: American Society of Anesthesiologists

## References

[1] Andre T, Boni C, Navarro M, Tabernero J, Hickish T, Topham C, Bonetti A, Clingan P, Bridgewater J, Rivera F, de Gramont A (2009). Improved overall survival with oxaliplatin, fluorouracil, and leucovorin as adjuvant treatment in stage II or III colon cancer in the MOSAIC trial. J Clin Oncol.

[2] National Comprehensive Cancer Network (2016). National comprehensive cancer network guidelines, Colon cancer (Version 2.2016). National Comprehensive Cancer Network.

[3] Taal BG, Van Tinteren H, Zoetmulder FA (2001). Adjuvant 5FU plus levamisole in colonic or rectal cancer: improved survival in stage II and III. Br J Cancer.

[4] Wolmark N, Rockette H, Fisher B, Wickerham DL, Redmond C, Fisher ER, Jones J, Mamounas EP, Ore L, Petrelli NJ (1993). The benefit of leucovorin-modulated fluorouracil as postoperative adjuvant therapy for primary colon cancer: results from National Surgical Adjuvant Breast and Bowel Project protocol C-03. J Clin Oncol.

[5] Hershman D, Hall MJ, Wang X, Jacobson JS, McBride R, Grann VR, Neugut AI (2006). Timing of adjuvant chemotherapy initiation after surgery for stage III colon cancer. Cancer.

[6] Andre T, Quinaux E, Louvet C, Colin P, Gamelin E, Bouche O, Achille E, Piedbois P, Tubiana-Mathieu N, Boutan-Laroze A, Flesch M, Lledo G, Raoul Y (2007). Phase III study comparing a semimonthly with a monthly regimen of fluorouracil and leucovorin as adjuvant treatment for stage II and III colon cancer patients: final results of GERCOR C96.1. J Clin Oncol.

[7] Bayraktar UD, Chen E, Bayraktar S, Sands LR, Marchetti F, Montero AJ, Rocha-Lima CM (2011). Does delay of adjuvant chemotherapy impact survival in patients with resected stage II and III colon adenocarcinoma?. Cancer.

[8] Berglund A, Cedermark B, Glimelius B (2008). Is it deleterious to delay the start of adjuvant chemotherapy in colon cancer stage III?. Ann Oncol.

[9] Czaykowski PM, Gill S, Kennecke HF, Gordon VL, Turner D (2011). Adjuvant chemotherapy for stage III colon cancer: does timing matter?. Dis Colon Rectum.

[10] Lima IS, Yasui Y, Scarfe A, Winget M (2011). Association between receipt and timing of adjuvant chemotherapy and survival for patients with stage III colon cancer in Alberta, Canada. Cancer.

[11] Xu F, Rimm AA, Fu P, Krishnamurthi SS, Cooper GS (2014). The impact of delayed chemotherapy on its completion and survival outcomes in stage II colon cancer patients. PLoS One.

[12] Yu S, Shabihkhani M, Yang D, Thara E, Senagore A, Lenz HJ, Sadeghi S, Barzi A (2013). Timeliness of adjuvant chemotherapy for stage III adenocarcinoma of the colon: a measure of quality of care. Clin Colorectal Cancer.

[13] Zeig-Owens R, Gershman ST, Knowlton R, Jacobson JS (2009). Survival and time interval from surgery to start of chemotherapy among colon cancer patients. J Registry Manag.

[14] Bos AC, van Erning FN, van Gestel YR, Creemers GJ, Punt CJ, van Oijen MG, Lemmens VE (2015). Timing of adjuvant chemotherapy and its relation to survival among patients with stage III colon cancer. Eur J Cancer.

[15] Klein M, Azaquoun N, Jensen BV, Gogenur I (2015). Improved survival with early adjuvant chemotherapy after colonic resection for stage III colonic cancer: a nationwide study. J Surg Oncol.

[16] Massarweh NN, Haynes AB, Chiang YJ, Chang GJ, You YN, Feig BW, Cormier JN (2015). Adequacy of the National Quality Forum’s colon cancer adjuvant chemotherapy quality metric: is 4 months soon enough?. Ann Surg.

[17] Nachiappan S, Askari A, Mamidanna R, Munasinghe A, Currie A, Stebbing J, Faiz O (2015). The impact of adjuvant chemotherapy timing on overall survival following colorectal cancer resection. Eur J Surg Oncol.

[18] Peixoto RD, Kumar A, Speers C, Renouf D, Kennecke HF, Lim HJ, Cheung WY, Melosky B, Gill S (2015). Effect of delay in adjuvant oxaliplatin-based chemotherapy for stage III colon cancer. Clin Colorectal Cancer.

[19] Sun Z, Adam MA, Kim J, Nussbaum DP, Benrashid E, Mantyh CR, Migaly J (2016). Determining the optimal timing for initiation of adjuvant chemotherapy after resection for stage II and III colon cancer. Dis Colon Rectum.

[20] Brezden-Masley C, Polenz C (2014). Current practices and challenges of adjuvant chemotherapy in patients with colorectal cancer. Surg Oncol Clin N Am.

[21] Fisher B, Gunduz N, Coyle J, Rudock C, Saffer E (1989). Presence of a growth-stimulating factor in serum following primary tumor removal in mice. Cancer Res.

[22] Harless W, Qiu Y (2006). Cancer: a medical emergency. Med Hypotheses.

[23] Baum M, Demicheli R, Hrushesky W, Retsky M (2005). Does surgery unfavourably perturb the “natural history” of early breast cancer by accelerating the appearance of distant metastases?. Eur J Cancer.

[24] Hensler T, Hecker H, Heeg K, Heidecke CD, Bartels H, Barthlen W, Wagner H, Siewert JR, Holzmann B (1997). Distinct mechanisms of immunosuppression as a consequence of major surgery. Infect Immun.

[25] Biagi JJ, Raphael MJ, Mackillop WJ, Kong W, King WD, Booth CM (2011). Association between time to initiation of adjuvant chemotherapy and survival in colorectal cancer: a systematic review and meta-analysis. JAMA.

[26] Des Guetz G, Nicolas P, Perret GY, Morere JF, Uzzan B (2010). Does delaying adjuvant chemotherapy after curative surgery for colorectal cancer impair survival? A meta-analysis. Eur J Cancer.

[27] Cheung WY, Neville BA, Earle CC (2009). Etiology of delays in the initiation of adjuvant chemotherapy and their impact on outcomes for Stage II and III rectal cancer. Dis Colon Rectum.

[28] Kim IY, Kim BR, Kim YW (2015). Factors affecting use and delay (>/=8 weeks) of adjuvant chemotherapy after colorectal cancer surgery and the impact of chemotherapy-use and delay on oncologic outcomes. PLoS One.

[29] Kim YW, Kim NK, Min BS, Lee KY, Sohn SK, Cho CH (2009). The influence of the number of retrieved lymph nodes on staging and survival in patients with stage II and III rectal cancer undergoing tumor-specific mesorectal excision. Ann Surg.

[30] von Elm E, Altman DG, Egger M, Pocock SJ, Gotzsche PC, Vandenbroucke JP, Initiative S (2007). The Strengthening the Reporting of Observational Studies in Epidemiology (STROBE) statement: guidelines for reporting observational studies. Lancet.

[31] Korean Academy of Medical Science (2012). Korean clinical practice guideline for colon and rectal cancer v.1.0. (Seoul: Korean Academy of Medical Science).

[32] Kim YW, Kim IY (2013). The role of surgery for asymptomatic primary tumors in unresectable stage IV colorectal cancer. Ann Coloproctol.

[33] Charlson ME, Pompei P, Ales KL, MacKenzie CR (1987). A new method of classifying prognostic comorbidity in longitudinal studies: development and validation. J Chronic Dis.

